# Polyhydroxyalkanoate synthesis by Sinorhizobium meliloti drives a host-specific collapse in symbiosis with Medicago sativa

**DOI:** 10.21203/rs.3.rs-7715224/v1

**Published:** 2025-10-29

**Authors:** Barney Geddes, Garrett Levin, Chinh Luu, Natalie Visich, Scott Hoselton, Anna Lipzen, Shuang Zhao, Liang Li, George diCenzo, Turlough Finan

**Affiliations:** North Dakota State University; North Dakota State University; North Dakota State University; North Dakota State University; North Dakota State University; Lawrence Berkeley National Laboratory; University of Alberta; University of Alberta; McMaster University

**Keywords:** Symbiosis, Nitrogen Fixation, Genetics

## Abstract

Naturally occurring root-nodule bacteria (rhizobia) vary substantially in their effectiveness at promoting growth of different plant hosts via symbiotic nitrogen fixation. These variations in rhizobial partner quality have important implications for the productivity of nitrogen-fixing symbioses in natural and agricultural ecosystems, yet we have a limited understanding of the genetic basis for this variation. In a case of host-specific reduction in symbiotic effectiveness (N_2_-fixation) with *Medicago sativa*, we identified the causative genetic elements from the pSymA replicon of *Sinorhizobum meliloti* HM006 and show them to be involved in polyhydroxyalkanoate (PHA) production in nitrogen-fixing bacteroids. Transfer of this gene region to a strain that forms an effective symbiosis with *Medicago sativa* resulted in a complete loss of symbiotic N_2_-fixation. We showed the mechanism for symbiotic collapse is the diversion of succinate semialdehyde pools in the bacteroid to gamma-hydroxybutyrate (GHB) by an iron-containing dehydrogenase, GhbD. These findings reveal unexpected impacts of carbon metabolism changes in nodules on symbiont performance and provide a rare example of mechanism for variation in rhizobium partner quality, suggesting that host-specific metabolic incompatibility may be a key player in the variations in partner quality observed in nature.

## Introduction

As the primary sustainable source of nitrogen nutrition in agricultural and natural ecosystems, the nitrogen-fixing root nodule symbiosis between rhizobia and legumes is a central player in global biogeochemical cycling and sustainable agricultural productivity. Variation in the symbiotic capacity of rhizobia is widespread within symbionts that are able to nodulate and fix nitrogen with a given host legume (1–3). Locally adapted native strains that are less effective at fixing nitrogen for their hosts limit the contribution of the symbiosis to agricultural systems by outcompeting elite inoculant strains for nodulation, dubbed “the rhizobium competition problem” (4–6). Though mechanisms remain elusive, hosts partially overcome this variation by selectively rewarding or punishing (sanctioning) rhizobia based on their productivity (7–10). Uncovering the genetics responsible for variation in microbial services to their hosts and elucidating what forces (e.g., “cheating”) maintain such widespread variation in symbioses despite host sanctioning mechanisms have emerged as key questions in microbial ecology (3). Recent studies point towards much of this variation being associated with mobile modular units such as plasmids and integrative conjugative elements (ICE) that are common features of rhizobium genomes (1, 11, 12).

In the tripartite genome of the model rhizobium *Sinorhizobium meliloti*, the “symbiotic megaplasmid” pSymA contains many essential genes for symbiosis with its *Medicago* hosts (*nod, nif* and *fix*). These gene sets, which encode the ability to produce the “Nod Factor” that initiates nodulation of the host (the *nod* genes) and to fix nitrogen in root nodules (the *nif* and *fix* genes) (13), account for ~ 5% of its size, yet are sufficient for functional symbiosis in the absence of the remaining ~ 95% of pSymA (14). The chromid pSymB is more ancestral than pSymA and has been implicated in playing a primary role in saprophytic competence (15), while also carrying the dicarboxylate transport genes (*dct*) essential for fueling nitrogen fixation in the nodule and exopolysaccharide synthesis (*exo*) genes important for plant invasion (16, 17). Despite decades of genetic study, the vast majority of genes on these replicons remain undefined with respect to their contributions to symbiosis. In part, this is due to the challenging nature of performing genetic screens for more subtle phenotypes (e.g., partner quality or competition for nodule occupancy) than the nodule-minus phenotypes of *nod* mutants or nitrogen-fixation-minus phenotypes of *nif* and *fix* mutants (18, 19), or of genes associated with symbiotic incompatibility (20, 21).

The rapid increase in genome sequencing of rhizobium isolates in recent decades has revealed that rhizobia, such as *S. meliloti*, contain large, open pangenomes (11, 22), further demonstrating the unexplored genetics that could contribute to variation in symbiosis outcomes. This wealth of genomic data, coupled with next-generation sequencing approaches to screen rhizobium symbiosis traits *en masse* (23, 24), enable the prediction of candidate genes that may impact the mutualism using quantitative genetics such as genome-wide association studies (GWAS) (12, 25–27). However, thus far, few candidates identified by these studies have been empirically validated for their role in symbiosis.

Here, we used strains wherein extrachromosomal replicons have been cured (15, 28) as surrogate hosts to identify genetic determinants that define differences observed in the vast diversity of effectiveness of functional symbioses between *Sinorhizobium* strains and their *Medicago* hosts. First, we investigated the co-transfer of partner quality with extrachromosomal replicons from five natural strains. The pSymA replicons were found to have the greatest impact on symbiotic performance and in the most extreme case, the transfer of pSymA from strain HM006 resulted in a substantial reduction of symbiotic performance with *Medicago sativa*. Next, through a series of refined deletions and gain-of-function experiments, the locus responsible for limiting symbiotic nitrogen fixation was identified and shown to be dominant. Genes within the locus were found to be involved in carbon polymer synthesis during symbiosis, and their impact on symbiotic N_2_-fixation revealed an unanticipated aspect to carbon metabolism in root nodules and its impact on partner quality.

## Results

### Co-transfer of host-specific symbiotic effectiveness phenotypes with the Sinorhizobium replicons.

We hypothesized that most genes contributing to variance in partner quality would be present on the extrachromosomal replicons in sinorhizobia and reasoned we could identify key genes involved in partner quality by transferring the extrachromosomal replicons (~ 40% of genome) to a surrogate *S. meliloti* background in which pSymA, pSymB, or both pSymA and pSymB were removed (14, 15, 29, 30). For replicon transfer, we used four genome-sequenced *S. meliloti* strains: KH46, HM006, USDA1021, and T073 and one *Sinorhizobium medicae* strain, WSM419 (31, 32). These genome-sequenced strains showed significant variation in partner quality (shoot-dry weight accumulation under nitrogen-limiting conditions) with *Medicago sativa* and *Medicago truncatula* (33) (**Supplemental Figure S1**).

**Figures 1:**
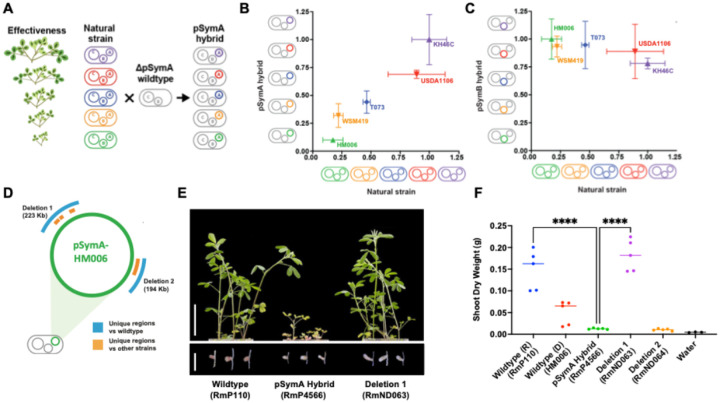
Delineating a Genetic Region Leading to Ineffective Symbiosis with Alfalfa. (**A**) Simplified scheme showing the transfer of pSymA from natural strains into a wildtype strain cured of its own pSymA. (**B**) Comparison between the relative abilities of natural strains versus their corresponding pSymA hybrid to promote shoot dry weight production by *M. sativa*. (**C**) Comparison between the relative abilities of natural strains versus their corresponding pSymB hybrid to promote shoot dry weight production by *M. sativa*. (**B and C**) Data are expressed as relative shoot dry weight compared to the mean maximum shoot dry weight of the best performing strain. (**D**) Diagram highlighting unique regions present in the HM006 pSymA replicon and absent in the pSymA of the wildtype RmP110 (blue) and other strains (KH46C, T073, USDA1106, and WSM419) (orange). (**E**) Representative images of *M. sativa*shoots and nodules inoculated with wildtype, pSymA hybrid, and Deletion 1. Scale bar represents 5 cm for the shoot panel and 0.5 cm for the nodule panel. (**F**) Quantitation of shoot dry weight accumulation by *M. sativa*.

The pSymA or pSymB replicons from the five strains were transferred independently or together to derivatives of the long-studied wildtype chromosomal background SU47 (Rm1021-derived background strain RmP110, or Rm5000) (33, 34) (Fig. 1A). The symbiotic productivity of the resulting hybrid strains with *M. sativa* revealed a clear positive correlation between the abilities of the natural strains to promote plant growth, and the relative abilities of hybrid strains containing their respective pSymA replicons (Fig. 1B, **Supplemental Figures S2A and S3A**). In contrast, no significant difference in plant growth promotion was observed for pSymB transfer (Fig. 1C, **Supplemental Figures S2A and S3A**). With *M. truncatula* as a host, increased growth relative to the wildtype was also observed by transfer of pSymAs from more effective strains (2- to 3-fold increase in SDW) and was enhanced with co-transfer of pSymB (~ 6-fold increase in SDW) (**Supplemental Figures S2B and S3B**).

### A genetic determinant on the S. meliloti HM006 pSymA leads to a collapse of symbiotic efficiency with M. sativa.

As a platform to investigate genetics that underly variation in pSymA partner quality, we chose the pSymA that conferred the most dramatic phenotype; a collapse in the productivity of the symbiosis with *M. sativa* conferred by the pSymA from *S. meliloti* HM006 when transferred to the RmP110 background, which forms an effective symbiosis with *M. sativa* when bearing its native pSymA. We first compared the pSymA contents of HM006 and RmP110. While known symbiosis gene sets in the pSymAs were nearly identical (**Supplemental Fig. 4**), two large regions from HM006 pSymA were found to be absent from the RmP110 pSymA (Fig. 1D, **Supplemental Figure S5**). Deletion of these regions from the HM006 pSymA-hybrid strain revealed that the loss of 223 kb in one region (Deletion 1, D1) completely restored symbiotic effectiveness with *M. sativa*, whereas the deletion of the second region (Deletion 2, D2) had no effect (Figs. 1E and 1F). Neither deletion significantly impacted symbiosis with *M. truncatula* (**Supplemental Figure S6**). We concluded that dominant genetic determinant(s) within the D1 region of the HM006 pSymA led to the collapse in effective symbiosis with *M. sativa*.

### Additive inhibition of symbiotic effectiveness by a duplicated gene cluster on HM006 pSymA.

**Figure 2:**
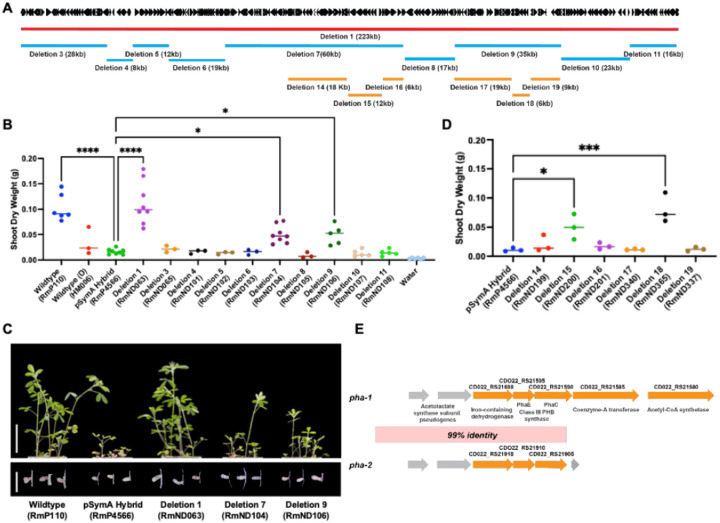
Relative locations and symbiotic effectiveness phenotypes of sub-deletions spanning the 223 kb region removed in D1. (**A**) Map displaying the relative locations of genes and approximate lengths of deletions across the region. (**B**) Quantitation of shoot dry weight accumulation by *M. sativa* following inoculation with the wild type, the pSymA hybrid, D1, and the sub-deletions of D1: D3–D11. (**C**) Representative shoot and nodules images of the partial recovery phenotype resulting from Deletions 7 and 9. Scale bars represent 5 cm for shoot and 0.5 cm for nodule images. (**D**) Quantitation of shoot dry weight accumulation by *M. sativa* following inoculation with the HM006 pSymA hybrid, and sub-deletions of D7 (D14-D16) and D9 (D17-D19). (**E**) Genetic diagram of the duplicated region found in both D15 and D18 and the annotations of predicted open reading frames within the putative operons.

To identify genes within the 223 kb region removed in D1 responsible for reduced symbiotic N_2_-fixation with *M. sativa*, we generated nine sub-deletions and screened them for their symbiotic productivity with *M. sativa* (Figs. 2A and 2B). While none of the deletions fully restored symbiotic productivity, two sub-deletions, D7 and D9, each resulted in a ~ 50% recovery in shoot dry weight production (Figs. 2A, 2B, and 2C). Further sub-deletions within the 60 kb region removed in D7 (D14-D16) and the 35 kb region removed in D9 (D17-D19) localized the genes responsible for the partial recovery of *M. sativa* shoot dry weight production to two gene sets deleted in D15 and D18 (Figs. 2A and 2D). Both genetic loci, which we name *pha-1* (removed in D15, locus tags CDO22_RS21580-CDO22_RS21610) and *pha-2* (removed in D18, locus tags CDO22_RS21900-CDO22_RS21925), show an operon-like configuration with a 99.9% identity to one-another over a majority of their length (Fig. 2E). Both clusters included genes annotated as encoding an iron-containing alcohol dehydrogenase (TIGR02638 E-value 1.70e-60) and two subunits of a Class III polyhydroxyalkanoic acid synthase: PhaE (TIGR01834 E-value 1.33e-08) and PhaC (TIGR01836 E-value 2.94e-113/3.25e-85) in an operon-like configuration. Homology between the loci extends 2783 bp upstream of these gene sets and contains two annotated pseudogenes along with, presumably, the promoter. A putative operon in *pha-1* extends beyond the duplicated region and included genes predicted to encode a Coenzyme A transferase (TIGR02428 E-value 8.25e-11) and an acetyl-CoA synthetase (TIGR02188 E-value 0).

### Symbiotic carbon polymer synthesis leads to a host-specific inhibition of productive symbiosis with M. sativa.

**Figure 3:**
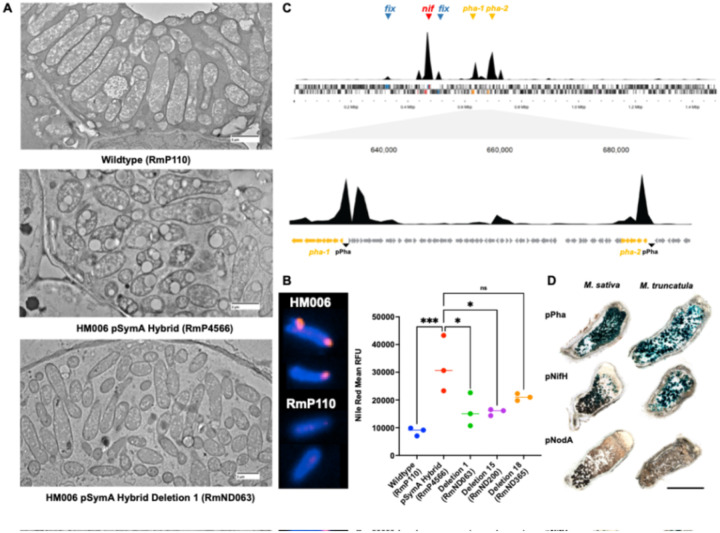
Symbiotic PHA production caused by the newly discovered operons in Region 15 and Region 18. (**A**) Examples of wildtype (RmP110) bacteroids in *M. sativa* nodules void of PHA in the top panel; examples of HM006 pSymA hybrid (RmP4566) bacteroids with an abundance of PHA granules in the middle panel; and examples of Deletion 1 (RmND063) bacteroids devoid of PHA granules in the bottom panel. Scale bar at 2 μm. (**B**) Quantification of PHA production by Nile Red staining of bacteroids collected from *M. truncatula* nodules. Data expressed as mean Nile Red relative fluorescence units (RFU). Images of Nile Red and DAPI fluorescence of representative isolated bacteroids are shown on left. (**C**) Transcription profile (RNAseq count) across the complete HM006 pSymA replicon (in HM006 pSymA hybrid strain) in bacteroids from nodules of *M. sativa*. The *fix* and *nif* genes are marked to show relative expression levels with respect to genes in *pha-1* and *pha-2*. Orange highlighted genes represent the *pha-1* and *pha-2* cluster. (**D**) GUS promoter fusion expression in *M. sativa* and *M. truncatula* root nodules. Depicted are images of nodules of plants inoculated with the HM006 pSymA hybrid strain (RmP4566) carrying *gus* downstream of the pPha promoter region, the pNifH promoter region (bacteroid-on positive control), or the pNodA promoter region (bacteroid-off negative control). Scale bars depict 100 μm.

Based on their annotation, we hypothesized that the gene sets in *pha-1* and *pha-2* may be involved in the production of a polyhydroxyalkanoate (PHA) carbon polymer such as polyhydroxybutyrate (PHB) during symbiosis. PHB is visualized by transmission electron microscopy (TEM) as large granules within bacteroids of rhizobia that synthesize PHB during symbiosis (35). TEM of *M. sativa* and *M. truncatula* nodules revealed the presence of large granules in the strains bearing pSymA-HM006. These granules were absent from bacteroids of the wild type or the D1 strain lacking both *pha-1* and *pha-2* (Fig. 3A, **Supplemental Figure S7**). While the small inefficient *M. sativa* nodules restricted further characterization, we also measured PHA production in effective *M. truncatula* nodules using flow cytometry, using PHA-bound Nile Red for quantitation (36) (Fig. 3B). A significant increase in Nile Red fluorescence of bacteroids was observed in wildtype HM006 and the HM006-pSymA hybrid strains relative to wildtype RmP110 (Fig. 3B). The increased fluorescence was significantly reduced in D1, which lacked both *pha-1* and *pha-2*, as well as D15 which lacked the expanded gene set of *pha-1* (Fig. 3B).

To examine the expression of the PHA synthesis genes during symbiosis, we analyzed the transcriptome of the HM006 pSymA hybrid strain in *M. sativa* and *M. truncatula* root nodules. RNAseq showed a high level of transcription across the regions that include the PHA synthesis genes in both hosts (Fig. 3C, **Supplemental Figure S8**). Several genes within them were among the top 50 most highly expressed genes from HM006 pSymA during symbiosis (**Supplemental Table S10**). We further observed robust *pha* cluster gene expression in nitrogen-fixing bacteroids of nodules using *gusA* promoter fusion constructs (Fig. 3D, **Supplemental Figure S9**). Altogether, these data indicate that the production of carbon polymers during symbiosis by bacteroids containing the newly discovered PHA genes drives a host-specific symbiotic incompatibility between *S. meliloti* and *M. sativa*.

### Expression of the pha genes alone leads to a complete symbiotic collapse in a naïve background that is relieved by local catabolism genes.

**Figure 4:**
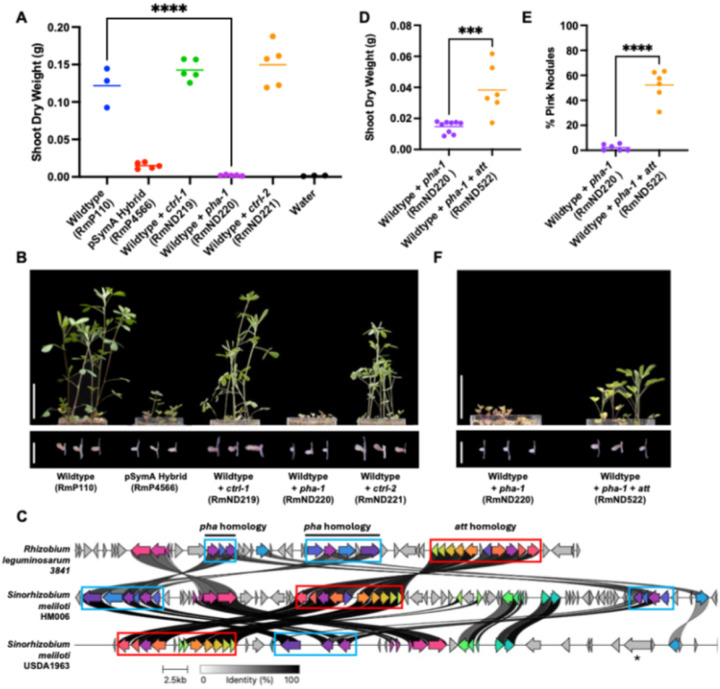
Expression of *pha* synthesis genes in a naïve background leads to a collapse in *M. sativa* symbiosis that is alleviated by a local catabolism gene set. (**A and D**) Quantitation of shoot dry weight accumulation and (**E**) percent of pink (Fix +) nodules produced by *M. sativa* following inoculation with the wild type, pSymA hybrid, or the wild type with various regions from HM006 pSymA integrated into the genome. *ctrl-1* and *ctrl-2,* respectively, represent the addition of Region 14 and Region 16 to the wild type. (**B and F**) Representative shoot and nodule images of shoot dry weight and nodule phenotypes from gain-of-function experiments in A, D, and E. Scale bars represents 5 cm in shoot pictures and 0.5 cm in nodule pictures. (**C**) Alignment of *S. meliloti* HM006, USDA1963 and *Rhizobium leguminosarum* 3841 *pha* homolog-containing genomic regions (Min. alignment sequence identity 0.3), visualized with clustermap.js (>0.5 alignment sequence identity shown). The * indicates *hrrP* gene encoding a known NCR peptidase (39).

To independently verify that the genes present in the *pha* loci are the sole components of the HM006 pSymA replicon responsible for the reduced symbiotic N_2_-fixation in *M. sativa* nodules, we applied a gain-of-function approach. We integrated the putative symbiotic PHA synthesis operon from *pha-1* (Figs. 2A and 2E) into the genome of RmP110 with its native pSymA. Strikingly, RmP110 with *pha-1* alone resulted in a more complete collapse of symbiosis than that associated with pSymA-HM006 (Fig. 4A). Shoot dry weight production was similar to uninoculated control plants (Fig. 4A), with few pink nodules (Fig. 4E) and barely detectable acetylene reduction activity (**Supplemental Figure S10**). TEM analysis showed that nodules were sparsely populated with bacteroids which showed signs of degradation by the host plant (37) (**Supplemental Figure S11**).

To rationalize the discordance between the partially reduced symbiotic efficiency conferred by the complete HM006 pSymA, and the near non-functional symbiosis rendered by the *pha* gene cluster alone, we hypothesized that other genetic determinants on HM006 pSymA alleviate the inhibition of symbiosis caused by the *pha* cluster. In search of such determinants, we identified other strains bearing *phaC* homologues by BLAST (**Supplemental Table S11**) and compared the content of the local genetic region surrounding *phaC* (Fig. 4C). These included the well-studied pea symbiont *Rhizobium johnstonii* (formerly *Rhizobium leguminosarum*) Rlv3841 as well as *S. meliloti* USDA1963. We found that a set of carbon metabolism genes previously characterized in *A. tumefaciens* C58 as involved in N-acylhomoserine lactone catabolism (*attKLM*) were consistently collocated with the *pha* cluster in other strains (38) (Fig. 4C), and we considered they could be responsible for alleviating the inhibition of symbiosis caused by the *pha* cluster alone when they were transferred with HM006 pSymA. We cloned the catabolism gene set and introduced them to the wild type that contained the *pha* gene cluster. Introduction of the catabolism genes was sufficient to restore functional symbiosis (Fix + phenotype) to the wildtype background characterized by increased shoot dry weight and pink nodule number (Figs. 4D, 4E, and 4F). Consistent with these data, Deletion 8 of HM006 pSymA which lacked these genes was more symbiotically impaired than other deletions (Fig. 2B).

### Evidence for a novel symbiotic poly(gamma)hydroxybutyrate cycle.

**Figure 5:**
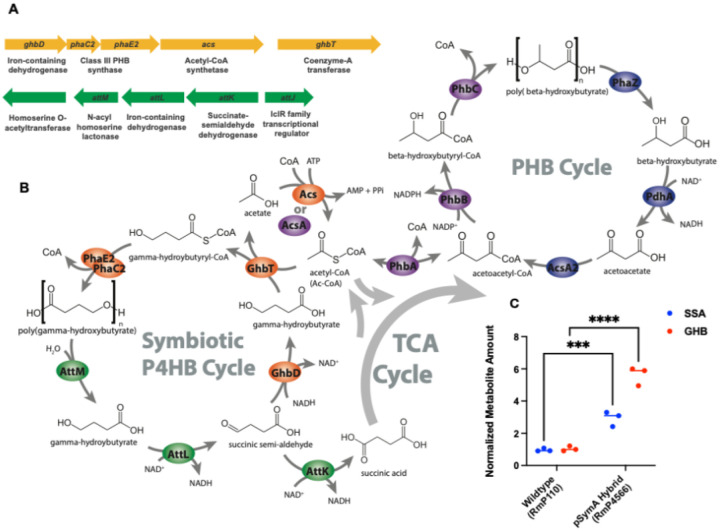
Proposed symbiotic P4HB cycle encoded by *S. meliloti* HM006 pSymA. (**A**) Diagram and annotations of conserved gene clusters associated with *pha* synthesis genes. (**B**) Proposed P4HB synthesis and catabolism pathways in the context of central metabolism and the well-characterized P3HB cycle in rhizobia. (**C**) LC-MS signal intensities for succinate-semialdehyde and gamma-hydroxybutyrate in *M. sativa* nodules occupied by wildtype or pSymA hybrid strains.

We considered that the recovery of symbiosis mediated by the catabolism genes may be the result of a PHA cycle, wherein the *pha* synthesis genes produce a polymer, whereas the *att* catabolism genes are able to break it down. Based on the annotation of the function of homologues to the *att* genes in *A. tumefaciens* (Fig. 5A), we predicted a novel pathway for polymer synthesis and catabolism based on the intermediate gamma-hydroxybutyrate (Fig. 5B). The proposed pathway places the synthesis genes in a rational scheme for polymer production based on their annotations; oxidation of succinate semialdehyde to gamma-hydroxybutyrate by the iron-containing dehydrogenase, the addition of an acetyl-CoA group by the CoA transferase generating GHB-CoA (with acetyl-CoA substrate generated by acetyl-CoA synthetase), and polymerization of GHB-CoA monomers via the PhaE/PhaC PHB synthase. The breakdown of the P4HB product of the proposed synthesis pathway then fits with the characterized activities of homologues to AttL and AttK, wherein they break down GHB to succinate, fueling the TCA cycle and bacteroid nitrogen-fixation. In this scheme, the lactonase AttM could conceivably resolve monomers from a GHB polymer with a similar hydration reaction to the cleaving of the lactone ring in gamma-hydroxybutyrolactone (38). To provide evidence for the proposed pathway, we performed metabolomics of root nodules formed by rhizobia bearing or lacking these pathways, looking for the presence and abundance of pathway intermediates. Consistent with the pathway scheme above, we found significant increase of both the predicted pathway intermediates, SSA and GHB (Fig. 5C, **Supplemental Figure S12**).

### Conversion of succinate semialdehyde to gamma-hydroxybutyrate by an iron-containing dehydrogenase in bacteroids is the mechanism for symbiotic collapse in M. sativa.

**Figure 6:**
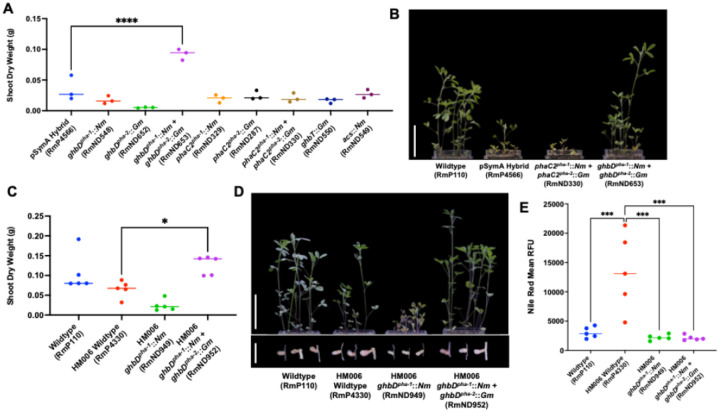
Removing *ghbD* from the *pha* loci results in a recovery of symbiosis with *Medicago sativa*. (**A and C**) Quantitation of shoot dry weight accumulation by *M. sativa* following inoculation with the RmP110 wildtype, HM006 wildtype, pSymA hybrid, or knockout strains in the pSymA hybrid (**A**) or HM006 (**C**) backgrounds. (**B and D**) Representative images of shoot dry weight production from the experiments in A and D. (**E**) Quantification of PHA production by Nile Red staining of bacteroids collected from *M. sativa* nodules. Data expressed as mean Nile Red Height.

To gain further insight into the role of individual genes in the pathway, we constructed knockout mutations within genes from each step of the predicted pathway (*ghbD*, *phaC2*, *ghbT*, and *acs*), with double knockouts created for the duplicated genes *ghbD* and *phaC2*. Strikingly, only a double knockout within the iron-containing dehydrogenase *ghbD* (the predicted first step of the synthesis pathway) was able to recover symbiosis (Figs. 6A and 6B). These data suggested that the first step of the predicted pathway, which we predicted to direct carbon from SSA away from succinate in a reductant consuming reaction, rather than polymer synthesis per se, was responsible for symbiotic collapse. To ensure these data were not an artifact of working in the HM006 pSymA hybrid background, we also constructed double-knockouts of the iron-containing dehydrogenase in HM006 and showed a similar recovery of symbiosis with *M. sativa* (Figs. 6C and 6D), coincident with a reduction in carbon polymer synthesis observed by flow cytometry (Fig. 6E, **Supplemental Figure S13**).

**Figure 7:**
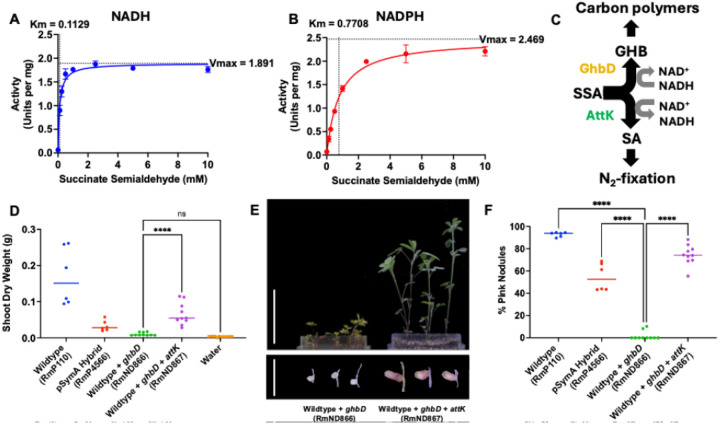
NADH-dependent reduction of succinic-semialdehyde by GhbD causes a collapse in symbiosis with *Medicago sativa*. (**A and B**) Enzyme activity based on colorimetric reduction of NADH (A) or NADPH (B) at 340 nM with purified GhbD and succinate semialdehyde. (**C**) Schematic of the outcomes of flux from SSA via GhbD or AttK enzymes. (**D**) Quantitation of shoot dry weight accumulation by *M. sativa* following inoculation with wildtype RmP110, the pSymA hybrid, or RmP110 expressing either *ghbD* or *ghbD* with *attK* from the native *ghbD*promoter. (**E**) Representative shoots and nodules of *M. sativa* plants. Scale bar represents 5 cm in shoot panel and 0.5cm in nodule panel. (**F**) The proportion of pink nodules from the experiment in D.

Since GhbD, the iron-containing dehydrogenase, appeared the critical element of symbiotic collapse, we wished to verify its hypothesized activity in the predicted P4HB synthesis pathway (Fig. 5B). We purified the iron-containing dehydrogenase and demonstrated its activity with SSA as a substrate (**Supplemental Figures S14 and S15**). Purified GhbD showed a Km of 0.1129 or 0.7708 and a Vmax of 1.891 or 2.469 in an NADH/NADPH linked spectrophotometric assay when incubated with SSA and NADH or NADPH, respectively, indicating a higher affinity for NADH as a cofactor (Figs. 7A and 7B). The loss-of-function data combined with the verified activity of GhbD suggested that the primary mechanism for symbiotic collapse is the biochemical conversion of SSA to GHB in bacteroids rather than polymer synthesis, since *phaC* double mutants retained symbiotic collapse phenotype (Figs. 6A and 6B). To further support this presumption, we expressed the dehydrogenase in the naïve wildtype background, without the downstream portions of the operon predicted to be involved in GHB polymerization (Figs. 7D to 7F). We found this construct was still sufficient for the collapse of *M. sativa* symbiosis previously observed by transferring the whole operon (Fig. 4). Based on the confirmed biochemical activity of GhbD (Figs. 7A and 7B), we further predicted we could rescue the symbiotic phenotype caused by *ghbD* expression by overexpressing *attK* from the catabolism gene set which in *Agrobacterium* is involved in the conversion of SSA into succinate in a reductant generating reaction (38) (Fig. 7C). Such an activity would compete with the conversion of SSA to GHB in a reductant-consuming reaction by GhbD (Fig. 7C). Indeed, we found that including the *attK* gene downstream of *ghbD* in the gain-of-function construct significantly restored symbiosis between *S. meliloti* and *M. sativa* (Figs. 7D to 7F).

## Discussion

Replicon transplantation experiments led to the finding that the synthesis of GHB-based hydrocarbon polymers is linked to the collapse of symbiotic effectiveness in *M. sativa* nodules leading to a reduction in partner quality. Several differences are notable between the carbon polymer production genes discovered here (Fig. 4H) and the well-characterized polyhydroxybutyrate synthesis genes (*phbABC*). First, annotations of genes in the operon (e.g., the inclusion of an iron-dependent dehydrogenase) are inconsistent with those in the well-characterized PHB synthesis pathway in rhizobia (40). Second, the high level of expression of these genes during symbiosis contrasts with the typically described pattern of PHB synthesis in *S. meliloti*, where PHB is produced in the free-living state and during infection thread invasion but not during symbiotic nitrogen fixation (36, 41). The inclusions in bacteroids observed in electron micrographs did not show the typical bright granules due to a lack of staining by lead citrate as is often observed for PHB (35). Finally, the observation of GHB accumulation in *M. sativa* nodules occupied by rhizobia containing the new *pha* synthesis genes (Fig. 5C), and the verification of GhbD as a dehydrogenase that reduces SSA to GHB (Fig. 7) further highlight the unique nature of this pathway. Altogether, these data support its existence as a novel symbiotic PHA synthesis pathway in rhizobia. We further supply evidence that the *att* catabolism genes impact the symbiotic phenotype associated with symbiotic PHA synthesis and propose that the *pha* and *att* clusters act together in a symbiotic P4HB cycle (Fig. 5B). A likely source of SSA, the substrate for the first step in the proposed synthesis pathway, is gamma-aminobutyrate (GABA) catabolism, which is provided to bacteroids from plant cells and catabolised to SSA via highly redundant transamination reactions (42–44).

It has been suggested that the production of reduced carbon storage polymers such as PHB may negatively impact the symbiosis by diverting resources away from nitrogen fixation (7). If the *M. sativa* phenotypes we document in this study was the result of diversion of resources from symbiosis via PHA synthesis, they would be by far the most dramatic observation of this phenomenon to date. To investigate the plausibility of this, we utilized an available host/symbiont metabolic model (VINE (45)) to investigate the degree to which the reduction in *M. sativa* partner quality could be explained by resource consumption (either reductant or carbon) by symbiotic PHA synthesis (**Supplemental Fig. 16**). While the modelling was consistent with resource consumption negatively impacting nitrogen fixation, the magnitude of reduction in shoot dry weight accumulation predicted by the model was significantly less than observed in *M. sativa* experimentally. We thus conclude that the observed symbiotic collapse cannot be explain simply due to diversion of reductant or carbon away from nitrogen fixation.

Instead, we hypothesize that the accumulation of GHB intermediates during PHA polymer synthesis in *M. sativa* nodules is toxic to *Sinorhizobium* bacteroids, resulting in a loss of nitrogen fixation capacity. This is supported by multiple observations. First, only mutations in the *pha* loci that abolish *ghbD* function enable the recovery of symbiosis in *M. sativa* (Fig. 6A). Similarly, expressing GhbD alone (converting SSA to GHB) in a naïve background resulted in a substantial reduction in symbiosis in *M. truncatula*, which was partially rescued by co-expressing AttL which diverts SSA to succinate (**Supplemental Figure S18**). Both of these observations suggest that synthesis of the pathway intermediates (rather than the polymer itself) was sufficient to collapse the symbiosis. Lastly, even though large PHA granules accumulate in bacteroids within both *M. sativa* and *M. truncatula* nodules, a substantial increase in GHB and SSA intermediates was only observed in *M. sativa* nodules (**Supplemental Figure S17**), consistent with our observation of a strong symbiotic phenotype only in this host.

The production of reduced carbon polymers such as PHB have been implicated in rhizobium “cheating”, in which the rhizobium benefits (storage of carbon for use once released from the nodule) at the expense of the host (reduced rate of nitrogen fixation) (7). However, it is not clear whether carbon polymer synthesis is a cheating behavior. Although PHB mutants showed increased nitrogen fixation in some rhizobia (46), limited effects were observed in others (35, 41, 46, 47). In fact, PHB production and other lipogenesis represent important reductant stores during symbiotic nitrogen fixation and therefore may facilitate rather than limit nitrogen-fixation capacity (48). In this study, removing the *pha* gene sets from HM006 pSymA did not significantly impact partner quality with *M. truncatula* (**Supplemental Figure S6**), even though the bacteroids within *M. truncatula* nodules did accumulate large PHA granules (Figs. 3B and 3D, **Supplemental Figures S7, S8 and S9**), suggesting that the polymer synthesis was not at the expense of the host. While P4HB synthesis in bacteriods came at a large cost to *M. sativa*, the proposed toxicity of the intermediates to the bacteriods and the complete collapse of the symbiosis likely means that P4HB synthesis hurt, rather than benefited, *S. meliloti*. Collectively, these results suggest that at least on the tested plants, P4HB synthesis is unlikely to represent a cheating behavior.

Presumably, the *pha* clusters confer some benefit to the rhizobia and/or the host to maintain them in rhizobium populations despite the potential for limiting partner quality on some hosts. PHA clusters have been theorized to benefit the symbiosis by providing a source of carbon to fuel SNF during carbon-limited scenarios (49), and the cycle we propose here would be particularly well suited to this purpose by engaging with central metabolism through dicarboxylates (unlike the canonical PHB catabolism pathway (50)), which must allow SNF in the nodule to drive ammonium excretion to the plant (51). PHAs accumulated in nodules could also benefit rhizobia outside of the nodule where they often encounter carbon-limited soil environments (36, 51). However, as bacteriods are terminally differentiated in *Medicago* nodules and thus cannot regrow in the soil, it may be that PHAs accumulated in bacteriods do not benefit free-living rhizobia. Alternatively, selection for maintenance of the *pha* clusters may be independent of the symbiosis, while their expression in bacteriods is non-beneficial and the result of inappropriate activation following detection of plant-produced signals.

Though the benefits conferred by the *pha* clusters to the microsymbiont and/or host remain to be elucidated, we expect that coevolution of these clusters in rhizobia with specific hosts can tune their activity to limit their negative impact on symbiosis, for example, through ensuring rapid flux through toxic pathway intermediates. Indeed, HM006 was isolated from *M. truncatula* nodules grown with soil from its native range (33, 52). When HM006 is present in *M. truncatula*, although the genes are highly expressed, we observes different gene expression patterns across the region relative to HM006 bacteroids in *M. sativa* nodules (**Supplemental Figure S8**), which may reflect the ability to fine tune the pathways through adaptation of transcriptional control. In addition, other genetic elements in the loci may participate in limiting the impact to specific hosts (Fig. 4C).

Partner quality differences in nature are widespread in many if not all rhizobium/legume systems (53–55). The replicon transplantation experiments described in this report revealed a correlation of partner quality phenotypes between *M. sativa* and the pSymA replicons of the tested strains. Thus pSymA (and perhaps the mobile symbiotic element in many rhizobia) likely represents the primary reservoir for accessary genes influencing partner quality in *Sinorhizobium*, although accessory genes on pSymB or additional plasmids can also influence symbiotic outcomes (21, 56). Given the much higher degree of genetic variation on extrachromosomal replicons relative to the chromosome (11, 22, 32, 57, 58), it is unsurprising these elements contain important determinants of the partner quality differences observed across different strains. Certainly, many mechanisms exist for the diversity in partner quality phenotypes observed between rhizobia and their different hosts, distributed through a reshuffling of genetic modules that engender metabolic flexibility but also can render individual partnerships inefficient (12, 59). This study suggests that along with diversity in symbiotic and immune signaling (60–63), metabolic incompatibility may be a key theme governing the diversity of these interactions we observe in nature.

## Materials and Methods

### Genetic Manipulations

Bacterial strains and plasmids used in this work are listed in **Supplemental Table S1**. Media used for routine growth and selection of microbes included LB for *Escherichia coli*, LBmc for *S. meliloti*, and YPD or SC defined media for *Saccharomyces cereviciae*. Antibiotics or supplements were added at routine concentrations when appropriate and details are summarized in the **Supplemental Methods**.

We routinely used a triparental mating protocol to transfer plasmids between donor and recipient strains by conjugation using the *E. coli* helper strain MT616 to drive mobilization (16). Transfer of extrachromosomal replicons between *S. meliloti* strains also utilized conjugation; either via an integrated *oriT* with triparental mating, or through the use of *rctB* in the donor strain to drive biparental conjugation (1). Deletions were made by FLP-*FRT* recombination (64), wherein *FRT* sites were integrated flanking a target region by homologous recombination, and subsequent excision of the region was catalyzed by introduction of FLP recombinase. Alternatively, region capture from *S. meliloti* involved a quadriparental mating with *S. meliloti* donor, *E. coli* helper, *E. coli* with a FLP-recombinase plasmid, and an *E. coli* recipient. FLP-*FRT* recombination was also used to introduce gene clusters into the genome by catalyzing recombination between an *FRT* site in a plasmid containing the cluster and a single *FRT* site in a landing pad in the genome (14). We used Φ M12 bacteriophage to transfer insertions between *S. meliloti* backgrounds using transduction (49). Selection of desired events involved the use of antibiotics as well as prototrophic selection. Further details of genetic manipulations are included in **Supplemental Methods.** Verification of genetic modifications was performed by PCR using primers in **Supplemental Tables S4 to S7** or by whole genome sequencing.

### Plasmid Construction

Plasmids for homologous recombination were routinely assembled using Level 1 (BsaI) Golden Gate cloning into suicide destination vectors that follow the BEVA architecture (66). Inserts were PCR amplified with primers found in **Supplemental Table S3.** Plasmids for gene cluster integration were assembled by yeast recombineering as previously described (66, 67). Primers for amplification of regions that were combined in the assembly are in **Supplemental Table S8.** Further details of plasmid construction are available in **Supplemental Methods.** Plasmids were verified by restriction digest or whole plasmid sequencing.

### Symbiotic Assays

Plant materials used in this work included *Medicago sativa* cv. Iroquois (alfalfa) and *Medicago truncatula* A17 (barrel medic). Plant assays were conducted in Leonard jar assembles with a 1:1 (wt:wt) mixture of sand and vermiculite with 250 mL of Jensen’s medium (68). Four to six seedlings were added to each Leonard jar and inoculated two days after with 10 mL of sterile water containing 100 μL of dense overnight culture. *M. sativa* seeds were surface sterilized for five minutes with 95% ethanol and for 20 minutes with 2.5% sodium hypochlorite. Seeds were then rinsed with sterile water for one hour, with water replaced every 15 minutes. *M. sativa* seeds were placed on 1.5% water agar, placed in the dark at room temperature, and allowed two days to germinate. *M. truncatula* seeds were first scarified with sulfuric acid for 10 minutes. After scarifying, seeds were rinsed 10 times with sterile water and then proceeded to be surface sterilized with 2.5% sodium hypochlorite for two minutes. Seeds were rinsed another 10 times with sterile water. *M. truncatula* seeds were placed on 1.5% water agar and placed in the dark at 4°C for three days. The seeds were then moved to room temperature and allowed two more days to germinate. Four to six seedlings were added to each Leonard jar and inoculated two days after with 10 mL of sterile water containing 100 μL of dense overnight culture.

Plants were grown in Conviron Gen1000/2000 growth chambers programmed with a day cycle of 18 hours at 21°C with maximum light followed by a night cycle of six hours at 17°C with no light. Plants were allowed 42 days post-inoculation to grow. After the 42-day period, shoots were cut from roots and placed in a drying oven for one week, after which they were weighed for shoot dry weight. Fix^+^ (pink) nodules were picked from roots, counted, and weighed immediately for average nodule fresh weight.

Collected nodules were placed in an air-tight container with desiccant packets and let dry for a week, after which they were weighed for nodule dry weight. In some experiments HP6890 gas chromatograph was used to perform gas chromatography for acetylene reduction assays as previously described (69). This assay was performed immediately after shoots were detached from the root system. A summary of all plant data collected is included in a **Supplemental Data** file.

### Flow Cytometry

Nodules pooled from four plants from one pot were macerated in 750 μL of phosphate-ascorbate buffer (PAB) using a mortar and pestle. An additional 750 μL of PAB was used to rinse and collect any additional bacteroids from the mortar. After vortexing briefly, samples were spun at 200g for 5 minutes to spin down residual plant debris. The resulting supernatant was then pelleted and resuspended in 100 μL of fixative (30% molecular-grade ethanol). After 15 minutes of incubation, samples were pelleted to remove the fixative and resuspended in 500 μL of PBS.

A portion of each sample was pooled to create the three control samples: unstained, DAPI-only, and Nile Red only. Prior to staining, samples were diluted 1:10 in PBS. 1 μL of 10% Nile Red in DMSO and 1 μL of DAPI (1 mg/mL in PBS) were added to samples and allowed 15 minutes to incubate in darkness. Following staining, samples were again diluted 1:10 in PBS, giving a final 1:100 sample dilution. Cells were analyzed on a Beckman Cytoflex S flow cytometer equipped with violet, yellow, red, and blue lasers. The 561 nm yellow laser was used to excite Nile Red, and fluorescence was detected using the 610/20 bandpass filter. DAPI was excited using the 405nm violet laser and detected with the 450/45 bandpass filter. DAPI-positive bacteroids were gated and analyzed for Nile Red fluorescence intensity. Details on the gating schemes are described in **Supplemental Figures S19 and S20**.

### Electron Microscopy

Specimens for transmission electron microscopy were fixed in 2.5% glutaraldehyde in sodium phosphate buffer, postfixed in 2% osmium tetroxide, dehydrated through a graded acetone series including saturated uranyl acetate in 70% acetone, and embedded in Spurr’s resin polymerized at 60°C for 24 hours. Embedded samples were sectioned at 70–90 nm thickness on an RMC MT XL ultramicrotome. Ultrathin sections were collected on copper grids with a formvar-carbon supporting film, then stained with Reynolds’ lead citrate. Observation and imaging were performed on a JEOL JEM-1400 electron microscope operating at 120 kV and equipped with an AMT NanoSprint 15L bottom-mount camera.

### GUS Staining and Microscopic Observations

The promoter regions of the *pha* clusters, *nifH*, and *nodA* were cloned via Golden Gate Cloning. Plasmids and strains used are listed in **Supplemental Table S1**. Primers used for promoter region amplification are listed in **Supplemental Table S3**. The nodules were fixed in 100 mM sodium-phosphate buffer with 0.1% Triton X-100 and then in 1.5% glutaraldehyde in 100 mM phosphate buffer under vacuum for 30 minutes and 90 minutes. After that, the nodules were embedded in 3% (w/v) low-melting agarose and sectioned into 100-μm slices using vibrating microtome VF-210–0Z (Precisionary, USA). The slices were then vacuum immersed in GUS Staining Solution for 30 min. The solution contains 2 mM 5-bromo-4-chloro-3-indoxyl-β-d-glucuronide cyclohexyl ammonium salt (X-Gluc), 0.2% Triton X-100, and 2 mM each of K3[Fe(CN)6] and K4[Fe(CN)6] in 50 mM sodium phosphate buffer (pH 7.2). After incubating at 37°C for 2 hours, the stained nodule slices were observed using a BioTek Cytation 5 Cell Imaging Multimode Reader (Agilent, USA).

### Enzyme Assays

A NADPH/NADH colorimetric assay was used to determine the Km and Vmax values of GhbD reacting with succinate semialdehyde (SSA). Individual 100 μL reactions comprised of purified GhbD (1 μg), NADPH (1 mM) or NADH (1 mM), SSA (0, 0.125, 0.25, 0.5, 1, 2.5, 5, or 10 mM), and reaction buffer (50 mM Na_3_PO_4_ pH 7.0, 200 mM NaCl, 10 mM MgCl_2_). The 340 nm absorbance value of every reaction occurring at 28°C was measured in 1-minute intervals for 11 minutes starting immediately after the addition of GhbD. Absorbance values were read using a BioTek Cytation 5 Cell Imaging Multimode Reader by Agilent. Simple linear regression tests were completed with absorbance values over time for specific substrate concentrations. The resulting slopes, with a R^2^ value > 0.95, were used to determine the rate of reaction per mg of GhbD present. These rates, at different substrate concentrations, were used in the Michaelis-Menten equation to determine the Km and Vmax values for GhbD with SSA and NADPH/NADH. GraphPad Prism Version 10.4.1 was used to perform simple linear regression and non-linear regression tests.

### Metabolomics

Metabolomics analysis of plant tissue samples was performed using a 4-channel chemical isotope labeling (CIL) LC-MS method (70). Normalization and labeling kits were obtained from Nova Medical Testing Inc. (Edmonton, AB, Canada). Each tissue sample was homogenized using a bead beater with steel beads, followed by the addition of ice-cold methanol/water (4:1, v/v) for metabolite extraction. The resulting extracts were normalized and labeled according to the protocols provided with the CIL kits.

LC-MS analysis was carried out using a Thermo Vanquish UHPLC system (ThermoFisher, Waltham, MA, US) coupled to a Bruker Impact II QTOF mass spectrometer (Bremen, Germany). Chromatographic separation was performed on an Agilent Eclipse Plus C18 reversed-phase column (150 × 2.1 mm, 1.8 μm particle size) maintained at 40°C. The mobile phases consisted of 0.1% (v/v) formic acid in water (A) and 0.1% (v/v) formic acid in acetonitrile (B). The gradient program was as follows: 0 min, 25% B; 10 min, 99% B; 15 min, 99% B; 15.1 min, 25% B; 18 min, 25% B. The flow rate was 400 μL/min. Mass spectra were acquired at 1 Hz over a mass range of m/z 220–1000. LC-MS data were processed using DataAnalysis (Bruker Daltonics) and IsoMS Pro (Nova Medical Testing Inc.). Manual verification of the key compound gamma-hydroxybutyrate (GHB) was performed using an authentic standard on an alternative instrumental platform. Specifically, MS/MS spectra and retention time (RT) data from the samples were compared to those of the standard, both acquired on a Dionex 3000 LC system coupled with Orbitrap Q Exactive HF mass spectrometer (ThermoFisher, Waltham, MA, US), using the same LC method and column. The MS/MS spectra of GHB in the samples, collected at multiple collision energies, matched those of the authentic standard, confirming the compound’s identity in the sample.

### DNA and RNA sequencing

Genome resequencing of hybrid strains and nodule RNA sequencing was performed by the Joint Genome Institute using Illumina NovaSeq technology. For RNA sequencing, bacteroids were isolated from nodules collected directly onto dry ice and flesh frozen in liquid nitrogen from one pot per replicate. RNA was extracted using a QIAgen RNeasy Plus Mini Kit, and underwent on-column DNase treatment. Libraries were prepared using Illumina’s Ribo-Zero rRNA removal kit and TruSeq Standard Total RNA HT sample prep kit. Raw fastq reads were filtered and trimmed according to the JGI QC pipeline, and mapped to the reference genome. Raw read counts were normalized to determine transcripts per million for each gene in the reference. Further details of DNA and RNA extraction, sequencing and bioinformatics analysis are included in **Supplemental Methods.** Raw data was deposited in NCBI SRA using accessions in **Supplemental Table S13.** Routine whole plasmid and whole genome resequencing to verify constructs and genetic manipulations was performed by Plasmidsaurus using Oxford Nanopore technology.

### Comparative Genomics

Analysis of homology between genes within clusters of interest was performed using clinker (Min. alignment sequence identity 0.3) and visualized with clustermap.js (> 0.5 alignment sequence identity shown) on the CAGECAT web server https://cagecat.bioinformatics.nl/ (71). Whole genome alignments and delineation of locally colinear gene blocks were performed using the MAUVE (72) plugin in the Geneious software with default parameters (automatically calculate seed weight and minimum LCB score with MUSCLE3.6 gapped aligner).

### Data Collection and Statistical Analysis

Plant experiments included uninoculated controls that were screened for the absence of nodules to confirm lack of contamination before any data collection. Individual replicates that makeup datapoints or were used for RNAseq or flow cytometry consisted of the average of all plants in one pot (six for *M. sativa* and four for *M. truncatula*). For statistical analysis we used a one-way ANOVA test with Dunnett’s multiple comparison test to establish significant differences between replicates. All data shown was significant as determined by one-way ANOVAs. In the figures, results from post hoc significance testing are indicated as *P < 0.0332, **P < 0.0021, ***P < 0.0002, ****P < 0.0001.

## Supplementary Files

This is a list of supplementary files associated with this preprint. Click to download.

- SupplementaryInformation2.0Final9.25.25.pdf
